# Initiation of antipsychotic medication among refugees, non-refugee migrants, second-generation migrants, and Swedish-born adults with incident non-affective psychotic disorders

**DOI:** 10.1007/s00127-025-02887-3

**Published:** 2025-04-03

**Authors:** Daniela Mellin, Ellenor Mittendorfer-Rutz, Christopher J. de Montgomery, Alexis E. Cullen, Heidi Taipale

**Affiliations:** 1 Department of Clinical Neuroscience, Division of Insurance Medicine, Karolinska Institutet, Stockholm, Sweden; 2 Department of Public Health, Danish Research Centre for Migration, Ethnicity and Health (MESU), University of Copenhagen, Copenhagen, Denmark; 3 Niuvanniemi Hospital, Kuopio, Finland; 4 School of Pharmacy, University of Eastern Finland, Kuopio, Finland

**Keywords:** Non-affective psychosis, Refugees, Antipsychotics, Initiation, Register-based cohort study, Sweden

## Abstract

**Background:**

It is not known if there are differences in antipsychotic initiation between migrants and native-born individuals diagnosed with non-affective psychotic disorder. This study aimed to determine (1) potential differences in initiation rate and type of first antipsychotic medication between refugees, non-refugee migrants, second-generation migrants, and Swedish-born young adults with incident non-affective psychosis and (2) which sociodemographic and clinical factors are associated with initiation.

**Methods:**

This register-based cohort included 12,960 adults aged 18–35 years, residing in Sweden during 2007–2018, with an incident diagnosis of a non-affective psychotic disorder in inpatient or specialised outpatient care. Sociodemographic and clinical factors associated with antipsychotic initiation were determined using modified Poisson regression models yielding Relative Risk, RRs, and 95% Confidence Intervals, CI.

**Results:**

Initiation of antipsychotic use was slightly less common among refugees (65.6%) compared to non-refugee migrants (70.2%), second-generation migrants (71.0%), and Swedish-born individuals (71.1%). However, after adjustment for sociodemographic and clinical factors, there was no difference in initiation rates between refugees and Swedish-born individuals (adjusted RR 0.96; 95% CI 0.92-1.00). Olanzapine was most common initial antipsychotic in all groups. However, compared to the Swedish-born, refugees (1.47; 1.10–1.97), non-refugee migrants (1.70; 1.26–2.27) and second-generation migrants (1.43; 1.05–1.97) were more likely to initiate the use with long-acting injectable antipsychotics, and also with first-generation antipsychotics, particularly haloperidol. Sociodemographic factors associated with initiation were similar among refugees and Swedish-born individuals, including younger age, higher education and inpatient care.

**Conclusion:**

Our finding that migrants were more likely to initiate long-acting antipsychotics suggests that clinical teams anticipate medication non-adherence among migrants.

**Supplementary Information:**

The online version contains supplementary material available at 10.1007/s00127-025-02887-3.

## Introduction

Non-affective psychotic disorder such as schizophrenia is a severe and debilitating mental illness, leading to impairments in several domains of everyday life [1]. There is consistent evidence showing that refugees have a higher risk of developing non-affective psychotic disorders than their host-country born peers [2]. This may be attributable to increased exposure to extreme stressors such as trauma, abuse and social isolation pre-, peri- and post-displacement [2, 3]. Additionally, refugees may face a lack of social support, discrimination, lower quality of life, stressful family situations, and distressing legal processes in gaining refugee status within their new host country which could also increase the risk for these disorders [1, 3, 4]. These same factors may also predispose refugees receiving less optimal care after illness onset, indeed, emerging evidence suggests that refugees with non-affective psychotic disorders are less likely to utilise psychiatric healthcare services and to use antipsychotics [5, 6].

Results of previous studies indicate that those who start their antipsychotic treatment early in the course of illness are more likely to achieve recovery compared to those who start later, and specifically, those who never start [7]. However, the knowledge base regarding possible differences in the initiation of antipsychotic medication between different migrant groups and the native population is limited. Previous studies have mainly assessed the frequency of antipsychotic dispensing in migrants in general [8, 9] or specific migrant groups [10]. Moreover, whilst second-generation migrants are also at greater risk of developing psychotic disorders compared to native populations (potentially due to acculturation difficulties), this group is seldom included in studies examining antipsychotic initiation and use [11].

Whilst several sociodemographic factors have been associated with non-adherence and discontinuation of antipsychotics in non-affective psychotic disorders information on initiation is lacking. The available results point towards younger age, low socioeconomic status such as lower educational level, and comorbid substance use disorder affecting antipsychotic non-adherence/discontinuation [12]. Furthermore, negative attitudes towards medication, poor insight, as well as adverse effects of medications appear to reinforce non-adherence and discontinuation of antipsychotics [13, 14]. Non-adherence is also associated with belonging to ethnic minority groups that may have experienced poor therapeutic alliance and/or barriers to care [12]. For instance, refugee youth are more likely to experience compulsory admission to psychiatric inpatient care, which has been identified as a risk factor for antipsychotic discontinuation [6], as well as to experience other coercive measures and use of physical restraints during psychiatric hospitalisation [15, 16]. Moreover, barriers to accessing good quality healthcare among refugees are often linked with socio-cultural attitudes, health illiteracy as well as linguistic barriers when communicating with healthcare professionals, leading to an increased risk of receiving inferior quality of care [15, 17]. Healthcare staff may also lack competence and knowledge in transcultural psychiatry, thereby delaying or compromising the quality of care [15]. Identifying factors associated with antipsychotic medication is crucial to developing pharmacological treatment regimens and targeted interventions to promote antipsychotic initiation. Here, it is important to investigate factors associated with initiation in refugees and non-refugee migrants specifically in order to ensure that these populations receive safe and effective treatments at an earlier stage of illness, potentially leading to better outcomes in these vulnerable groups.

The present study compared antipsychotic initiation among refugees and other migrant groups with incident non-affective psychotic disorders and investigated whether similar clinical and socioeconomic factors are associated with antipsychotic initiation or non-initiation in these groups. Given the sparsity of previous research investigating whether minority groups are more likely to initiate particular types of antipsychotic medication, we also compared medication types across these groups. Our aims were to determine whether there are differences in initiation and type of first antipsychotic medication between refugees, non-refugee migrants, second-generation migrants, and Swedish-born individuals diagnosed with incident non-affective psychotic disorder and to investigate whether sociodemographic and clinical factors are associated with initiation of antipsychotic use.

## Methods

### Study setting

This cohort study is based on nationwide registers in Sweden linking individual-level data via a unique (de-identified) personal identification number given to residents in Sweden either at birth or at immigration. Information regarding diagnosis and hospitalisations were derived from the National Patient Register, NPR, kept by the Board of Health and Welfare [18]. Additionally, data on medication use was derived from the Prescribed Drug Register, held by the Board of Health and Welfare, and sociodemographic variables were collected from Longitudinal Integration Database for Health Insurance and Labour Market Studies, LISA, held by Statistics Sweden [19]. Refugee status was identified from the longitudinal database for integration studies, STATIV, held by Statistics Sweden [20]. Micro Data for Analyses of Social Insurance (MiDAS) register provided data on sickness absences and disability pensions. Furthermore, dates of death were identified from the Cause of Death Register kept by the Board of Health and Welfare [21].

### Study population

The study population comprised all individuals in Sweden, aged 18–35 years, with incident diagnosis of non-affective psychotic disorder. Diagnoses were determined using International Classification of Diseases − 10th Revision (ICD-10) codes assigned in inpatient or specialised outpatient care. We included all individuals with ICD-10 codes F20-F29 recorded during 2007–2018. To capture incident cases, individuals were required to have no recorded inpatient/ outpatient contacts with a main diagnosis of non-affective psychotic disorder in the previous three years and to have resided in Sweden for at least three full calendar years prior to cohort entry. All individuals entered the cohort at the date of their first diagnosis of a non-affective psychotic disorder and were followed up through to 31 December 2018. To ensure that these were incident cases, a washout period for antipsychotic use was applied. Those individuals who had been dispensed antipsychotics 3–15 months before incident diagnosis were excluded from the study. Additionally, from 13 439 individuals, those who emigrated (*N* = 20) and those who died during first 6 months of diagnosis were excluded from these analyses (*N* = 88). Those individuals whose first contact was inpatient care, and whose duration of care exceeded 100 days, were excluded from the study (*N* = 371; 3.7%). This cut-off was chosen, since most inpatient care durations did not exceed 100 days, except very few cases with considerably long stays.

### Outcome

The primary outcome of this study was initiation of antipsychotic use (vs. not initiating), between 3 months before (T-3) and 6 months (T + 6) after the first psychosis diagnosis in inpatient or specialised outpatient care. Type of first initiated antipsychotic was also assessed.

Antipsychotics were defined according to the Anatomical Therapeutic Chemical (ATC) classification system as the code N05A (excluding lithium ATC code N05AN01) based on data derived from the Prescribed Drug Register. Antipsychotics were further categorised into oral medications (see Supplementary Table 1) vs. long-acting injectable antipsychotics (LAIs, grouped together). Those who were dispensed two or more antipsychotics on the same date were categorised as initiating antipsychotic polytherapy. Additionally, initial antipsychotics were categorised as first-generation vs. second-generation antipsychotics.

### Exposure

Immigration status was the main exposure, defined as refugee, non-refugee migrant, second-generation migrant, or Swedish-born, obtained from the STATIV database. Refugees were defined as immigrants whose grounds for residence were either refugee status, whether for humanitarian reasons or according to rights stipulated in the 1951 Refugee Convention [22]. Additionally, whether they arrived as asylum seekers or through the UN resettlement programme or family reunified to a refugee [23]. All others were classified as non-refugee migrants. Individuals born in Sweden with both parents born in Sweden were categorised as Swedish-born. Lastly, those individuals born in Sweden with at least one parent born outside of Sweden were classified as second-generation migrants.

### Covariates

An array of socioeconomic and clinical covariates was included in the analyses (detailed definitions are provided Supplementary Table 1). Sociodemographic factors were assessed on 31 December in the year preceding cohort entry and included sex, age (categorised as 18–23, 24–29, 30–35 years) at first contact, educational level (low, medium, high) and marital status (unmarried vs. married/registered partner vs. divorced). Measures of work disability and labour market marginalization included sickness absence (SA; 0 vs. 1–90 vs. >90 days), receipt of disability pension (DP) and registered unemployment (0 vs. 1-180 vs. >180 days) during the previous calendar year. In addition, for refugees and non-refugee migrants, we determined the duration of residency at first diagnosis (< 5 vs. 6–10 vs. >11 years) and country of birth (Afghanistan, Iraq, Iran, Somalia, Sweden, Former Yugoslavia, other Africa, other Europe, other Middle East, Asia, The Americas), as detailed in Supplementary Table 2.

Previous use of other psychotropic medications included antidepressants (ATC code N06A), benzodiazepines (N05BA, N05CD), z-drugs (N05CF), other anxiolytics (N05B excluding N05BA), other hypnotics (N05C excluding N05CD and N05CF), and mood stabilisers (carbamazepine N03AF01, valproic acid N03AG01, lamotrigine N03AX09, lithium N05AN01). Use of these psychotropic drugs were identified three months before the first diagnoses (T-3 to T0).

Clinical characteristics included whether a person was treated in inpatient/specialised outpatient care at first contact non-affective psychosis, and the type of diagnosis. The latter was categorised as schizophrenia/schizoaffective disorder (ICD-10 codes F20, F25), acute/transient psychotic disorders (F23), unspecified, nonorganic psychosis (F29), and other psychotic disorders (F21, F22, F24, F28). Previous psychiatric diagnoses received in inpatient/outpatient care were also categorised as affective psychosis (F3 disorders with psychotic features: F30.2, F31.2, F32.3, F33.3), anxiety disorders (F40-41), depression (F32-33), bipolar disorder (F30-31), disorders of adult personality and behaviour (F60-69), substance use disorders (F10-19), and previous suicide attempts (X60-X84 and Y10-Y34).

### Statistical analysis

Data management and statistical analyses were done using Stata, version 16.0, for Windows. Descriptive statistics for the study population were presented as percentages for categorical variables by immigration status and means with standard deviations (SD) for continuous variables. Sociodemographic and clinical factors associated with antipsychotic initiation (vs. no initiation) were analysed using modified Poisson regression models with robust error variance estimation and reported as unadjusted Relative Risks (RRs) and adjusted RRs (aRRs), with 95% confidence intervals (CIs) [24]. Subgroup analyses were conducted among refugees. Initial antipsychotic medications were analysed for refugees, non-refugee migrants and second-generation migrants compared to Swedish-born individuals, by adjusting for age, sex, type of first diagnosis and duration of first care period in inpatient care.

As certain study variables contained missing values, imputed values were used. Educational level was missing for 479 observations (< 3.7% of the total sample), these cases were assigned to the low education category (< 10 years) as it was deemed highly probable that the educational level of these individuals fell into this range. Seven cases with missing data for marital status were assigned to the unmarried category as this was most prevalent in the current sample. Additionally, one missing observation was recorded for the birth country, which was assigned to the Asia category.

## Results

### Sociodemographic and clinical characteristics

The sociodemographic characteristics of the studied groups, namely refugees *n* = 1581, non-refugee migrants *n* = 1368, second-generation migrants *n* = 1315 and Swedish-born *n* = 8696 are provided in Table 1. The final cohort included *N* = 12 960 individuals of whom men formed the majority 8426 (65.0%), comprising 71.5% of refugees, 56.3% of non-refugee migrants, 68.6% of second-generation migrants, and 64.7% of Swedish-born individuals. The mean (SD) age at cohort entry for the study cohort was 32 (4.96) years. Specifically, the mean age for refugees was 32 (6.09) years, 34 (6.29) years for non-refugee migrants, 30 (6.39) years for second-generation migrants, and 32 (6.13) years for Swedish-born young adults.

**Table 1:** Characteristics of the study cohort, comprising individuals diagnosed with first-episode non-affective psychotic disorder at baseline, by migration status

	Refugees *N* = 1581	Non-refugee migrants *N* = 1368	Second-generation migrants *N* = 1315	Swedish-born *N* = 8696	All *N* = 12,960
**Sociodemographic characteristics**					
Age at first contact					
18–23 years	35.4 (559)	21.7 (297)	47.3 (622)	37.3 (3247)	36.5 (4725)
24–29 years	36.2 (572)	33.0 (452)	29.8 (392)	36.6 (3186)	35.5 (4602)
30–35 years	28.5 (450)	45.2 (619)	22.9 (301)	26.0 (2263)	28.0 (3633)
Sex					
Women	28.5 (451)	43.7 (598)	31.4 (413)	35.3 (3072)	35.0 (4534)
Men	71.5 (1130)	56.3 (770)	68.6 (902)	64.7 (5624)	65.0 (8426)
Country of birth			NA	NA	
Afghanistan	5.4 (85)	0.3 (4)			0.7 (89)
Asia	3.2 (50)	21.8 (298)			2.7 (348)
F. Yugoslavia	18.0 (285)	4.2 (57)			2.6 (342)
Iraq	18.3 (289)	3.1 (42)			2.6 (331)
Iran	7.3 (116)	3.9 (53)			1.3 (169)
Other Africa	10.6 (168)	13.1 (179)			2.7 (347)
Other Europe	6.4 (101)	32.8 (449)			4.2 (550)
Other Middle East	9.4 (148)	6.9 (95)			1.9 (243)
Somalia	18.0 (284)	1.3 (18)			2.3 (302)
The Americas	3.5 (55)	12.7 (173)			1.8 (228)
Number of years in Sweden prior to the first contact			NA	NA	NA
3–5 years	19.3 (305)	23.8 (326)			
6–10 years	21.6 (342)	24.9 (341)			
>10 years	51.2 (701)	59.1 (934)			
Education					
Low (<10)	47.2 (702)	35.8 (462)	42.4 (538)	34.0 (2864)	36.6 (4566)
Medium (10–12)	14.1 (209)	15.0 (194)	9.7 (123)	9.5 (803)	10.7 (1329)
High (>12)	38.8 (577)	49.2 (636)	47.9 (607)	56.5 (4766)	52.8 (6586)
Marital status					
Unmarried	76.2 (1205)	70.3 (961)	91.8 (1207)	94.6 (8225)	89.5 (11598)
Married	16.7 (264)	19.9 (272)	4.7 (62)	4.1 (360)	7.4 (958)
Divorced	7.1 (112)	9.9 (135)	3.5 (46)	1.3 (111)	3.1 (404)
**Labour market factors**					
Unemployment during previous year					
0 days	55.4 (876)	65.9 (902)	67.3 (885)	71.2 (6195)	68.4 (8858)
1-180 days	34.0 (538)	25.7 (351)	27.1 (356)	23.4 (2032)	25.3 (3277)
>180 days	10.6 (167)	8.4 (115)	5.6 (74)	5.4 (469)	6.4 (825)
Sickness absence during previous year					
0 days	90.7 (1434)	88.0 (1204)	90.5 (1190)	85.4 (7423)	86.8 (11251)
1–90 days	5.3 (84)	7.8 (107)	5.6 (74)	8.3 (722)	7.6 (987)
>90 days	4.0 (63)	4.2 (57)	3.9 (51)	6.3 (551)	5.6 (722)
Disability pension at cohort entry	7.3 (116)	8.6 (117)	12.4 (163)	13.2 (1144)	11.9 (1540)
**Characteristics at first contact**					
Type of first contact					
Inpatient care	53.8 (850)	55.8 (763)	50.2 (660)	50.7 (4409)	51.6 (6682)
Outpatient care	46.2 (731)	44.2 (605)	49.8 (655)	49.3 (4287)	48.4 (6278)
Diagnosis at first contact					
Schizophrenia/ schizoaffective	8.7 (137)	7.5 (102)	6.9 (91)	8.1 (703)	8.0 (1033)
Acute and transient psychosis	34.9 (552)	35.9 (491)	31.0 (407)	38.0 (3305)	36.7 (4755)
Unspecified psychosis	46.4 (733)	45.7 (625)	52.0 (684)	42.6 (3700)	44.3 (5742)
Other psychosis	36.4 (575)	37.8 (517)	32.9 (432)	41.2 (3581)	39.4 (5105)
**Psychiatric comorbidity**					
Affective psychosis	2.3 (36)	2.4 (33)	1.8 (23)	2.8 (245)	2.6 (337)
Anxiety disorder	11.1 (176)	10.8 (147)	13.5 (178)	17.7 (1542)	15.8 (2043)
Bipolar disorder	1.6 (25)	1.7 (23)	2.4 (31)	3.2 (276)	2.7 (355)
Depression	10.3 (162)	9.9 (135)	11.0 (145)	15.3 (1327)	13.7 (1769)
Personality disorder	2.5 (40)	4.0 (55)	4.3 (57)	4.5 (392)	4.2 (544)
Substance use disorder	15.8 (250)	13.3 (182)	20.5 (269)	20.4 (1774)	19.1 (2475)
Previous suicide attempt	4.6 (73)	4.8 (65)	4.9 (64)	6.9 (597)	6.2 (799)
**Use of other psychotropic drugs at baseline**					
Antidepressants	11.5 (182)	12.9 (176)	13.2 (174)	20.7 (1797)	18.0 (2329)
Benzodiazepines	3.7 (59)	4.8 (66)	6.2 (81)	8.0 (692)	6.9 (898)
Z-drugs	6.8 (108)	7.7 (105)	7.5 (99)	11.2 (976)	9.9 (1288)
Non-benzodiazepine anxiolytics	5.8 (92)	6.2 (84)	7.0 (92)	8.0 (693)	7.4 (961)
Other hypnotics	5.3 (84)	5.9 (80)	5.9 (77)	7.7 (673)	7.1 (914)
Mood stabilisers	1.8 (28)	1.2 (17)	2.0 (26)	3.1 (272)	2.7 (343)

At cohort entry, 6682 (51.6%) individuals received inpatient care, while 6278 (48.4%) received specialised outpatient care. Among all four groups, unspecified psychosis and acute and transient psychotic disorders, were the most common first diagnoses. The most common previous psychiatric comorbidity was substance use disorder (19.1%), followed by anxiety disorder (15.8) and depression (13.7%). Antidepressants were the most used psychotropic drug among all groups before the first contact (18.0%), followed by Z-drugs (9.9%) and non-benzodiazepine anxiolytics (7.4%).

There was a higher prevalence of unemployment among refugees compared to the other groups; however, refugees were slightly less likely to have received sickness absence payments and disability pension compared to their non-refugee migrant, second-generation migrant, and Swedish-born peers. Moreover, 8.6% of refugees were diagnosed with schizophrenia/ schizoaffective disorder, compared to 7.5% of non-refugee migrants, 6.9% of second-generation migrants and 8.1% of Swedish-born young adults. The largest proportion of refugees (18.3%) were from Iraq followed by refugees from Former Yugoslavia (18.0%), whereas most non-refugee migrants (32.8%) were from other countries in Europe (Other Europe).

### Initiation of antipsychotic use

Table 2 provides the association between different factors and initiation of antipsychotic use. Overall, a total of 9120 (70.4%) individuals initiated antipsychotic medication between T-3 and T + 6 (for simplicity, referred to as ‘during first 6 months’). Proportion of initiation varied between the groups with 65.7% of refugees, 70.2% of non-refugee migrants, 71.0% of second-generation migrants, and 71.1% of Swedish-born found to initiate antipsychotic use within the first 6 months. In the unadjusted analysis, refugees (RR 0.92; 95% CI 0.89–0.96) were less likely than their Swedish-born peers to initiate antipsychotic medication. However, after adjustment for covariates, the difference between refugees and Swedish-born individuals was attenuated, and the association was no longer statistically significant (RR 0.96; 95% CI 0.92-1.00). Furthermore, there were no statistically significant differences in the risk of initiating antipsychotic use for either non-refugee migrants or second-generation migrants relative to Swedish-born individuals in the unadjusted model or the adjusted model.

**Table 2:** Unadjusted and adjusted modified Poisson models of the association between sociodemographic and clinical factors as well as migration status with antipsychotic initiation among young adults with incident non-affective psychosis. Relative risks (RRs) and 95% confidence intervals (CI). Adjusted for all factors listed below

	Non-initiators *N* = 3840	Initiators *N* = 9120	Unadjusted RR (95% CI)	Adjusted RR (95% CI)
Age at first contact				
18–23 years	34.3 (1317)	37.4 (3408)	ref	ref
24–29 years	36.3 (1395)	35.2 (3207)	0.97 (0.94–0.99)	0.94 (0.92–0.97)
30–35 years	29.4 (1128)	27.5 (2505)	0.96 (0.93–0.98)	0.93 (0.90–0.96)
Women	31.7 (1218)	36.4 (3316)	1.06 (1.04–1.09)	1.02 (1.00-1.05)
Group				
Swedish-born	65.3 (2509)	67.8 (6187)	ref	ref
Second-generation migrant	9.9 (381)	10.2 (934)	0.99 (0.96–1.04)	1.01 (0.97–1.05)
Non-refugee migrant	10.6 (407)	10.5 (961)	0.99 (0.95–1.02)	1.01 (0.97–1.05)
Refugee	14.1 (543)	11.4 (1038)	0.92 (0.89–0.96)	0.96 (0.92-1.00)
Education				
Low (<10)	42.3 (1571)	38.2 (2995)	ref	ref
Medium (10–12)	12.8 (474)	9.8 (855)	0.98 (0.94–1.03)	1.01 (0.96–1.05)
High (>12)	45.0 (1672)	57.0 (4914)	1.14 (1.11–1.17)	1.13 (1.09–1.16)
Marital status				
Unmarried	88.7 (3404)	89.9 (8194)	ref	ref
Married	7.2 (276)	7.5 (682)	1.01 (0.99–1.05)	1.03 (0.99–1.08)
Divorced	4.2 (160)	2.7 (244)	0.85 (0.79–0.93)	0.91 (0.84–0.98)
Unemployment during previous year				
0 days	65.3 (2509)	69.6 (6349)	ref	ref
1-180 days	27.0 (1038)	24.6 (2239)	0.95 (0.93–0.98)	0.97 (0.95-1.00)
>180 days	7.6 (293)	5.8 (532)	0.90 (0.85–0.95)	0.93 (0.89–0.98)
Sickness absence during previous year				
0 days	89.1 (3422)	85.8 (7829)	ref	ref
1–90 days	4.7 (181)	8.8 (806)	1.17 (1.14–1.21)	1.12 (1.08–1.16)
>90 days	6.2 (237)	5.3 (485)	0.97 (0.92–1.02)	0.95 (0.90-1.00)
Disability pension at cohort entry	12.1 (43)	11.8 (1077)	0.99 (0.96–1.03)	1.01 (0.97–1.05)
Type of first contact				
Inpatient care	44.8 (1721)	54.4 (4961)	ref	ref
Outpatient care	55.2 (2119)	45.6 (4150)	0.74 (0.73–0.75)	0.87 (0.85–0.89)
Diagnosis at first contact				
Acute and transient psychosis	9.9 (379)	7.7 (701)	ref	ref
Schizophrenia/ schizoaffective	8.5 (325)	7.8 (708)	1.06 (0.99–1.12)	1.04 (0.97–1.10)
Unspecified psychosis	40.1 (1540)	46.1 (4202)	1.13 (1.08–1.18)	1.10 (1.04–1.15)
Other psychosis	41.6 (1596)	38.5 (3509)	1.06 (1.01–1.11)	1.00 (0.96–1.06)
Affective psychosis	1.5 (59)	3.1 (278)	1.18 (1.12–1.24)	1.10 (1.05–1.16)
Anxiety disorder	14.6 (560)	16.3 (1483)	1.04 (1.02–1.07)	1.02 (0.99–1.06)
Bipolar disorder	2.0 (75)	3.1 (280)	1.17 (1.06–1.19)	1.08 (1.02–1.15)
Depression	12.8 (490)	14.0 (1279)	1.03 (1.00-1.06)	1.00 (0.97–1.04)
Personality disorder	5.1 (194)	3.8 (350)	0.92 (0.86–0.97)	0.93 (0.87–0.99)
Substance use disorder	23.0 (884)	17.5 (1591)	0.90 (0.87–0.92)	0.93 (0.90–0.96)
Previous suicide attempt	7.5 (286)	5.6 (513)	0.91 (0.86–0.96)	0.92 (0.87–0.97)
Antidepressants	14.3 (550)	19.5 (1779)	1.11 (1.08–1.14)	1.06 (1.03–1.09)
Benzodiazepines	5.4 (206)	7.6 (692)	1.10 (1.06–1.15)	1.03 (0.98–1.07)
Z-drugs	7.1 (274)	11.1 (1014)	1.13 (1.10–1.17)	1.08 (1.04–1.12)
Non-benzodiazepine anxiolytics	4.6 (176)	8.6 (785)	1.18 (1.14–1.21)	1.10 (1.07–1.14)
Other hypnotics	4.2 (160)	8.3 (754)	1.19 (1.15–1.23)	1.14 (1.10–1.18)
Mood stabilizers	2.3 (89)	2.8 (254)	1.05 (0.99–1.12)	1.01 (0.95–1.09)

In the adjusted analysis, factors associated with increased chances of antipsychotic initiation in the entire cohort were high educational level (RR 1.13; 95% CI 1.09–1.16, compared to low education), previous sickness absence of 1–90 days (RR 1.12; 95% CI 1.08–1.16, compared to 0 gross days) (Table 2) and previous diagnosis of affective psychosis (RR 1.10; 95%CI 1.05–1.16). Moreover, previous use of antidepressants (RR 1.06; 95% CI 1.03–1.09), Z-drugs (RR 1.08; 95% CI 1.04–1.12), non-benzodiazepine anxiolytics (RR 1.10; 95% CI 1.07–1.14), and other hypnotics (RR 1.14; 95% CI 1.10–1.18), were associated with increased likelihood of antipsychotic initiation.

On the other hand, there was a lower likelihood of antipsychotic initiation among older individuals (24–29 years RR 0.94; 0.92–0.97 and 30–35 years 0.93; 0.90–0.96, compared to 18–23 years), those with previous long-term unemployment (RR 0.93; 95% CI 0.89–0.98), those with outpatient care as first contact type (RR 0.88; 95% CI 0.86–0.90), those diagnosed with substance use disorder (RR 0.93; 95% CI 0.90–0.96), personality disorder (0.93; 0.87–0.99) and those with previous suicide attempts (RR 0.92; 95% CI 0.87–0.97).

### Migration-related factors

Factors associated with antipsychotic initiation were further assessed separately among refugees in subgroup analyses (Table 3). Factors associated with increased likelihood of antipsychotic initiation in refugees included high education level (RR 1.16; 95%CI 1.07–1.26), female sex (1.02; 1.00-1.05) and sickness absence of 1–90 days (1.18; 1.04–1.34) whereas long-term unemployment (0.84; 0.74–0.97) and being born in the Americas (0.75; 0.56–0.97) were associated with decreased risk. Time since arrival to Sweden was not associated with antipsychotic initiation.

**Table 3:** Subgroup analyses of sociodemographic, clinical and migration-related factors associated with antipsychotic initiation among refugees with incident non-affective psychosis. Unadjusted and adjusted modified Poisson regression models as relative risks (RR) and 95% confidence intervals (CIs). Adjusted for all factors listed in the table

	Unadjusted RR (95% CI)	Adjusted RR (95% CI)
Age at first contact		
18–23 years	ref	ref
24–29 years	1.00 (0.92–1.03)	1.00 (0.91–1.10)
30–35 years	1.01 (0.93–1.11)	1.03 (0.93–1.15)
Women	1.14 (1.06–1.23)	1.09 (1.01–1.18)
Education		
Low (< 10)	ref	ref
Medium (10–12)	0.87 (0.76-1.00)	0.88 (0.77–1.01)
High (> 12)	1.17 (1.08–1.26)	1.16 (1.07–1.26)
Unemployment during previous year		
0 days	ref	ref
1-180 days	0.87 (0.80–0.94)	0.91 (0.84–0.98)
>180 days	0.83 (0.72–0.95)	0.84 (0.74–0.97)
Sickness absence during previous year		
0 days	ref	ref
1–90 days	1.19 (1.05–1.34)	1.18 (1.04–1.34)
>90 days	0.93 (0.75–1.13)	0.89 (0.73–1.08)
Type of first contact		
Inpatient care	ref	ref
Outpatient care	0.95 (0.88–1.02)	0.94 (0.87–1.01)
Diagnosis at first contact		
Acute and transient psychosis	1.07 (0.91–1.27)	1.16 (0.98–1.38)
Schizophrenia/ schizoaffective	ref	ref
Unspecified psychosis	1.01 (0.89–1.16)	1.04 (0.90–1.20)
Other psychosis	1.01 (0.88–1.15)	1.01 (0.87–1.16)
Personality disorder	1.03 (0.83–1.28)	1.06 (0.85–1.32)
Substance use disorder	0.88 (0.79–0.98)	0.96 (0.85–1.06)
Previous suicide attempt	0.78 (0.63–0.98)	0.82 (0.65–1.03)
Country of birth		
Afghanistan	0.83 (0.68–1.01)	0.90 (0.73–1.09)
Asia	0.92 (0.74–1.15)	0.85 (0.67–1.07)
F. Yugoslavia	ref	ref
Iraq	0.95 (0.84–1.06)	0.97 (0.86–1.09)
Iran	1.02 (0.88–1.17)	1.04 (0.90–1.20)
Other Africa	0.88 (0.77–1.02)	0.87 (0.75–1.01)
Other Europe	1.01 (0.87–1.17)	1.03 (0.90–1.20)
Other Middle East	0.92 (0.80–1.07)	0.95 (0.82–1.10)
Somalia	0.97 (0.87–1.08)	0.99 (0.88–1.12)
The Americas	0.71 (0.53–0.93)	0.75 (0.56–0.97)
Number of years in Sweden prior to the first contact		
3–5 years	ref	ref
6–10 years	1.15 (1.02–1.29)	1.13 (1.00-1.28)
>10 years	1.07 (0.96–1.18)	0.98 (0.88–1.10)

### Type of first antipsychotic medication initiated

**Fig. 1:**
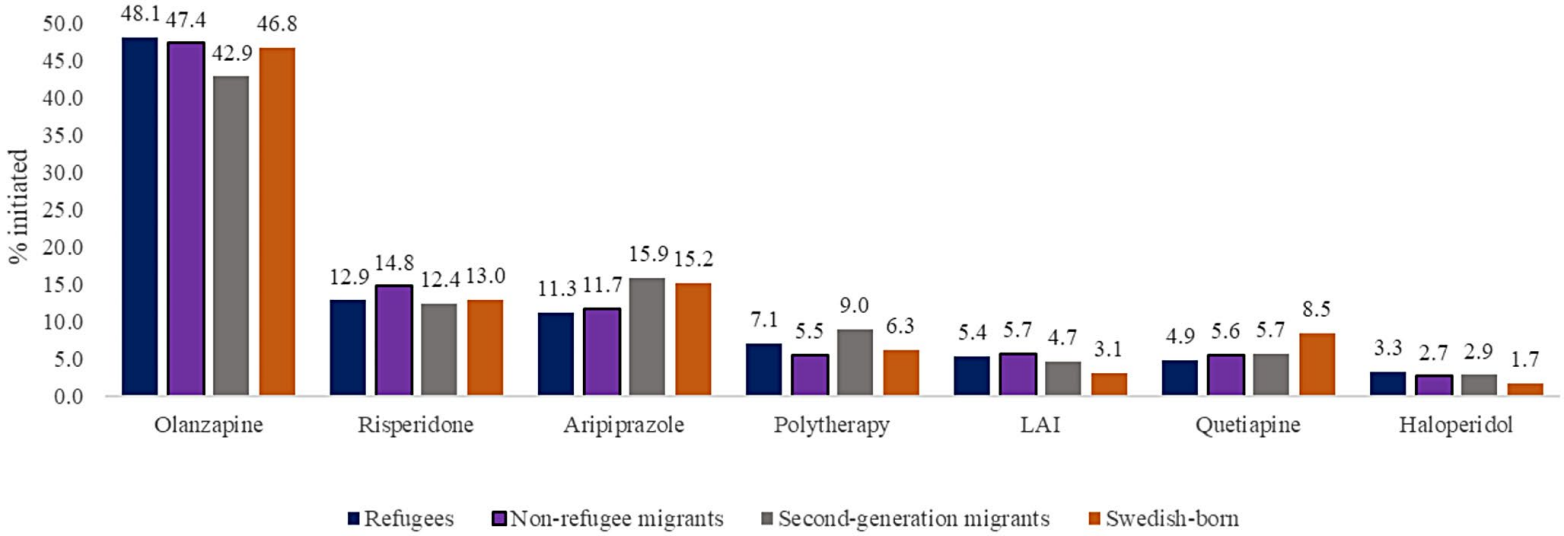
Most common first antipsychotics in refugee, non-refugee migrant, second-generation migrant and Swedish-born patients with incident non-affective psychosis

Type of antipsychotics initiated in the four studied groups are shown in Fig. 1. Oral olanzapine was the most frequently used initial antipsychotic (*n* = 4249; 46.6% of users), followed by oral aripiprazole (*n* = 1315), and oral risperidone (*n* = 1197) in all four groups. Antipsychotic polytherapy was prescribed for 4.6% (*n* = 598) and LAIs for 2.7% (*n* = 344) as their initial antipsychotic in outpatient care. Within the LAI category, the most common specific drug was risperidone LAI (*n* = 94), followed by olanzapine LAI (*n* = 56), zuclopenthixol LAI (*n* = 55), paliperidone LAI (*n* = 47) and aripiprazole LAI (*n* = 36).

Compared to Swedish-born young adults with incident non-affective psychosis, refugees (RR 1.47; 95%CI 1.10–1.97), non-refugee migrants (1.70; 1.26–2.27) and second-generation migrants (1.43; 1.05–1.97) were more likely to initiate LAIs, when adjusting for age, sex, type of first diagnosis and first care duration (Table 4). The same pattern was also observed for haloperidol where refugees (1.79; 1.21–2.62) and second-generation migrants (1.72; 1.13–2.61) were more likely to initiate haloperidol as their first antipsychotic medication. In contrast, refugees (0.69; 0.57–0.83) and non-refugee migrants (0.77; 0.64–0.92) were less likely to initiate aripiprazole and all migrant groups were less likely to initiate quetiapine (refugees 0.55; 0.42–0.73, non-refugee migrants 0.63; 0.48–0.84, second-generation migrants 0.89; 0.52-0.90) compared to Swedish-born individuals.

**Table 4:** Adjusted relative risks (RRs) and 95% confidence intervals (CI) of type of first antipsychotic medication initiated among refugees, non-refugee migrants, and second-generation migrants with incident non-affective psychosis. The reference is the Swedish-born category. LAI: long-acting injectable antipsychotic.

	Refugee	Non-refugee migrant	Second-generation migrant
Olanzapine	0.95 (0.88–1.03)	1.00 (0.92–1.08)	0.91 (0.84-1.00)
Risperidone	0.92 (0.77–1.09)	1.13 (0.95–1.34)	0.94 (0.78–1.13)
Aripiprazole	0.69 (0.57–0.83)	0.77 (0.64–0.92)	1.00 (0.85–1.17)
Polytherapy	1.03 (0.81–1.31)	0.90 (0.68–1.19)	1.37 (1.09–1.72)
LAI	1.47 (1.10–1.97)	1.70 (1.26–2.27)	1.43 (1.05–1.97)
Quetiapine	0.55 (0.42–0.73)	0.63 (0.48–0.84)	0.89 (0.52–0.90)
Haloperidol	1.79 (1.22–2.62)	1.46 (0.95–2.25)	1.72 (1.13–2.61)

Adjusted RR for sex, age, type of first diagnosis, first care duration

When initial antipsychotics were categorised as first-generation vs. second-generation antipsychotics, second-generation migrants (13.1%, *N* = 122, RR 1.05, 95% CI 1.02–1.07), refugees (12.3%, *N* = 128, RR 1.03, 95% CI 1.02–1.05) and non-refugee migrants (11.2%, *N* = 108, RR 1.02, 95% CI 1.00-1.04) were more likely to initiate first-generation antipsychotics than Swedish-born individuals (8.4%, *N* = 517).

## Discussion

The results of this nationwide register-based cohort study showed that refugees were somewhat less likely to initiate antipsychotic use compared to their Swedish-born peers. However, after adjustments for clinical and sociodemographic factors this difference was no longer observed. Oral olanzapine was the most frequent choice of first antipsychotic among all groups, followed by oral aripiprazole and oral risperidone. Compared to Swedish-born individuals, all migrant groups were more likely to initiate LAIs or oral haloperidol and less likely to initiate quetiapine as their first antipsychotic. Factors associated with increased likelihood of antipsychotic initiation in the total cohort were younger age, higher educational level, previous sickness absence of 1–90 days (compared to 0 gross days), and inpatient care at first contact. On the other hand, previous long-term unemployment and having previous diagnosis of substance use disorder or personality disorder, as well as previous suicide attempts were associated with decreased likelihood of antipsychotic initiation.

Overall, 70% of patients with incident non-affective psychosis initiated antipsychotic use. To the best of our knowlegde, we are not aware of studies assessing differences in initiation of antipsychotics for incident non-affective psychosis between migrant groups. However, our findings are partly aligned with a previous Swedish register-based cohort study, suggesting that refugees were less likely to use antipsychotics [25]. Although the previous Swedish study found differences in antipsychotic treatment patterns 2 years after non-affective psychosis diagnosis, these differences were attenuated after 5 years had passed. Similarly, a Finnish study by Lehti et al., found that migrants diagnosed with a psychotic disorder were less likely to initiate antipsychotic medication, and more likely to discontinue their medication regimens than their Finnish-born peers [6]. Specifically, 31.9% of migrants in the Finnish study did not initiate antipsychotic use, whereas corresponding non-initiation rate in our study was 34.4% among refugees and 29.8% among non-refugee migrants. However, the present study was able to distinguish between refugees and non-refugee migrants whereas the previous Finnish study combined these groups. In addition, the Finnish study was not restricted to incident cases. Although differences in initiation in this present study were observed between refugees and Swedish-born, they were no longer observed after adjustments for clinical and sociodemographic factors. Our interpretation is that factors such as age, educational level, labour market -related factors and psychiatric comorbidity explained the difference observed in the unadjusted analysis or that these factors are stronger predictors of initiation than migration status.

Interestingly, the type of first antipsychotic drug initiated differed between migrant groups although the same agents, namely oral olanzapine, aripiprazole and risperidone were most frequently initiated in all groups. Refugees, non-refugee migrants, and second-generation migrants had 40–70% higher likelihood of initiating LAIs as their first antipsychotic medication compared to Swedish-born. These differences persisted after adjusting for age, sex, type of first diagnosis and duration of first inpatient care. Similarly refugees and second-generation migrants had a higher likelihood of initiating first-generation antipsychotics, particularly haloperidol as their first antipsychotic medication compared to their Swedish-born peers. Our findings are broadly in line with studies from the USA, where ethnic minorities are more commonly prescribed older antipsychotics (such as haloperidol), compared to non-minority groups [26]. Use of haloperidol and other first-generation antipsychotics may be an indicator of potential disparities in prescribing practices which is consistent with other studies showing that migrant groups are more likely to have a first contact for psychosis via involuntary or emergency settings [27, 28]. More frequent initiation with LAIs may be a marker of concerns relating to non-adherence to antipsychotics which has been associated with minority groups who may have experienced barriers to care [12]. This finding is in line with that of a previous Swedish study reporting that LAI use was more prevalent among migrant populations than Swedish-born individuals [25]. Refugees were less likely to initiate their antipsychotic use with quetiapine or aripiprazole, medications which are associated with fewer adverse effects such as extrapyramidal symptoms and prolactin elevation and aripiprazole with less weight gain. Reasons behind the observed differences between migrant groups in initial choice of antipsychotic should be further examined.

A systematic review from 2016 found that lower age, lower educational level, substance use disorder, ethnic differences, lower quality of life, and experiencing barriers to care may be proxies for antipsychotic non-adherence, although findings for age at onset were inconsistent [12]. In the present study we observed a lower likelihood of antipsychotic initiation among those of higher age at first contact (20–24 or 30–35 years) compared to those of younger age (18–23 years). This may be attributable to younger individuals experiencing more severe symptoms and seeking medical help at an earlier stage of illness. In accordance with findings of the systematic review on non-adherence [12] and from another Swedish cohort study, the results of the present study showed that high educational level (>12 years) was associated with higher likelihood of antipsychotic initiation. Higher educational level may lead to greater knowledge of the healthcare system and better health literacy, yielding better outcomes in general and specifically also among refugees.

We found that sickness absence for 1–90 gross days was associated with higher likelihood of initiating antipsychotic use compared to those who had not experienced SA (0 gross days). This may describe individuals who have already exhibited symptoms causing them to be on sick leave from work in the preceding year, and only later they received a non-affective psychosis diagnosis. This implies that they may have been treated for depression or anxiety disorders, and treatments for these conditions were not effective. Lastly, in line with our previous work showing that unemployment was associated with lower prevalence of antipsychotic medication use [25], the current study found long-term unemployment to be associated with lower likelihood of antipsychotic initiation. One possible explanation for these findings is that unemployed individuals are less able to afford prescribed medication due to limited financial resources.

Several clinical factors were related to increased or decreased likelihood of initiating antipsychotic treatment. Individuals with comorbid substance use disorder, personality disorder or previous suicide attempt had decreased likelihood of antipsychotic. The relationship between schizophrenia and comorbid substance use disorder has been well reported [25], as this comorbidity often results in non-adherence and discontinuation of antipsychotic medication use [14, 29]. Extra attention should be paid to healthcare of this group, since previous studies have shown that antipsychotics are effective in preventing relapse also among persons with comorbid substance use disorder [30]. Furthermore, initiation of antipsychotic medication has been associated with decreased risk of developing a new substance use disorder among individuals diagnosed with non-affective psychosis. Decreased likelihood of antipsychotic initiation among persons with personality disorder may stem from their previous experiences of pharmacotherapy trials which often are unsuccessful in treating personality disorders such as borderline personality disorder [31]. On the other, individuals who had previously used other psychotropic drugs and/or had a previous affective psychosis or bipolar disorder diagnosis may have had regular contact with healthcare, yielding better outcomes, as suggested by previous research conducted in the USA [32].

Previous studies have observed that prior suicide attempts prior to illness onset are related to poor antipsychotic adherence [33]. This is line with the present study, where suicide attempts were associated with lower likelihood of antipsychotic initiation. This finding suggests that poor psychiatric health may act as a predictor for antipsychotic non-initiation. Moreover, inpatient care at first contact was positively associated with antipsychotic initiation. A possible explanation for this is that individuals may have experienced more severe symptoms, requiring inpatient care as first type of contact. However, it is concerning that persons with previous suicide attempts initiate antipsychotic treatment less often.

In our study, migration-related factors such as time since arrival to Sweden and country of birth did not play a major role in the associations with antipsychotic initiation. This is contrast to study by Lehti et al., [34] that reported lower likelihood of antipsychotic use among migrants born in the Middle East. In the present study, refugees from the Americas (mainly referring to Chile) had lower likelihood of antipsychotic initiation. Additionally, another Swedish register-based study found longer duration of stay to be a predictor for higher prevalence of psychotropic drug use [25] whereas that was not found in the present study. Possibly duration of stay in the host country plays a role in non-adherence secondary to initiation and they may benefit from closer monitoring during continuous medication use. Among refugees, women were more likely than men initiate antipsychotic use which may signal that refugee men may experience greater barriers to initiation.

### Strengths and limitations

This study had several methodological strengths, particularly owing to the comprehensive data derived from nationwide registers in Sweden. The registers provide individual-level information of high quality over extended periods, without loss to follow-up, reducing selection bias. Another strength lay in the fact that the nationwide registries provide access to an extensive range of sociodemographic and clinical factors, medication use, and data on outcomes. The results are generalisable to other countries akin to Sweden, with similar state-funded healthcare system providing care to all residents.

Nevertheless, this study had limitations. First, this study included individuals who had resided 3 years in Sweden prior to their first non-affective psychosis diagnosis (incident diagnosis) to ensure that they are experiencing their first psychotic episode (i.e. differentiate them from prevalent cases). Therefore, it excludes those individuals who developed non-affective psychotic disorder shortly after arrival. Second, the National Patient Register only includes inpatient and specialised outpatient care and excludes primary care visits. As individuals may have been treated in primary care before, we included individuals who initiated antipsychotic use at maximum of three months before an incident non-affective psychosis diagnosis. Third, the Prescribed Drug Register provides information on prescriptions dispensed by pharmacies, not containing information on prescriptions that remain undispensed. Consequently, it is not known whether some patients who did not initiate antipsychotic use were in fact prescribed antipsychotics. Fourth, missing data on education level may have led to an underestimate of individuals having a medium (10–12 years) or higher education (>12 years). However, the proportions of missing values were similar in all four groups, suggesting that any potential misclassification may be non-differential.

## Conclusions

In this register-based cohort study, refugees were somewhat less likely to initiate antipsychotic use compared to their Swedish-born peers, but the difference was not observed after adjustment for other factors. Although most initiated antipsychotics were similar between the groups, migrant groups had an increased likelihood of initiating LAIs and oral haloperidol as first antipsychotic medication compared to Swedish-born individuals. More common initiation with LAIs may indicate that migrants were presumed to have higher likelihood for medication non-adherence. Regarding non-initiation of antipsychotic use, extra attention should be paid to those with older age, lower educational level, to those with previous sickness absence, specialised outpatient care as first contact, comorbid substance use disorders and previous suicide attempts.

## Electronic supplementary material

Below is the link to the electronic supplementary material.

Supplementary Material 1: Supplementary Table 1 - Covariate definitions. Supplementary Table 2. Description of the categorization of birth countries used throughout the study.

## Data Availability

These data cannot be made publicly available due to privacy regulations. According to the General Data Protection Regulation, the Swedish law SFS 2018:218, the Swedish Data Protection Act, the Swedish Ethical Review Act, and the Public Access to Information and Secrecy Act, these types of sensitive data can only be made available for specific purposes, including research, that meets the criteria for access to this type of sensitive and confidential data as determined by a legal review. Readers may contact Professor Kristina Alexanderson (kristina.alexanderson@ki.se) regarding the data.
