# Supplementary material for: Initiation of antipsychotic medication among refugees, non-refugee migrants, second-generation migrants, and Swedish-born adults with incident non-affective psychotic disorders
Source: Soc Psychiatry Psychiatr Epidemiol. 2025 Apr 3;60(12):2773–84. doi: 10.1007/s00127-025-02887-3 (PMC12594656; doi:10.1007/s00127-025-02887-3)
Supplement: Supplementary file 1 — Supplementary Material 1: Supplementary Table 1 - Covariate definitions. Supplementary Table 2. Description of the categorization of birth countries used throughout the study. [file 127_2025_2887_MOESM1_ESM.docx]

**Supplementary Table 1 – Covariate definitions.**

| **Supplementary Table 1**. Covariate definitions | |
| --- | --- |
| **Variable** | **Definition (ICD-10 or ATC codes)** |
| Birth year | The year the individual was born |
| Sex | Sex (male/female) |
| Immigration age | The age at the year of immigration |
| Group | Defined as 0 = Swedish-born, 1 = second-generation migrant, 2 = non-refugee migrant, 3 = refugee |
| Educational level | Measured previous calendar year before diagnoses, categorised as low ≤ 9, medium 10–12, high > 12 years, or missing. Missing is combined with ‘low’ category |
| Family situation | Marital status during previous calendar year before diagnoses (married/unmarried/divorced). Missing are combined with unmarried |
| Country of birth | As defined in Supplementary Table 2 below. Missing is combined with Asia category |
| First year of contact | Defined as first day of diagnosis (starting point for this study) |
| First type of contact | Whether the individual had 1) inpatient, 2) outpatient care due to non-affective psychosis (main diagnosis F2) at the diagnosis or during 6 months after |
| First care duration | For those whose first contact was inpatient care, the number of days (restricted to 100). Zero for outpatients. |
| Duration of residency (for refugees and migrants) | Measured at diagnoses date (if possible) or during previous calendar year before diagnoses, categorised as ≤ 5 years, 6–10 vs. >10 years |
| Type of diagnosis, at baseline | Whether the individual had a diagnosis of 1) schizophrenia (F20) or schizoaffective disorder (F25), 2) acute and transient psychotic disorders (F23), 3) unspecified nonorganic psychosis (F29), 4) other psychosis, namely schizotypal disorder (F21), persistent delusional disorder (F22), induced delusional disorder (F24), other nonorganic psychotic disorders (F28), at the first diagnosis. |
| **Labour market variables** | |
| Unemployment | 1: 1-180 days, 2 = > 180 days vs. 0: no unemployment during the previous calendar year |
| Disability pension | Yes/no, measured at the date of first diagnosis |
| Sickness absence | 1: 1–90 gross days, 2: >90 gross days vs. 0 = no SA, measured one year before first diagnoses |
| **First antipsychotic dispensed** | Categorised as:  Olanzapine (N05AH03)  Quetiapine (N05AH04)  Risperidone (N05AX08)  Aripiprazole (N05AX12)  Haloperidol (N05AD01)  Long-acting injectable  Polytherapy = two or more antipsychotics dispensed at the same date  Other antipsychotic (next most common ones included Clozapine (N05AH02) , Perphenazine (N05AB03), Levomepromazine (N05AA02), Flupentixol (N05AF01))  Additionally, first-generation antipsychotics included categories of N05AA-N05AG, whereas second-generation antipsychotics included N05AH and N05AX. |
| **Psychiatric comorbidities based on inpatient and specialised outpatient care diagnosis,** measured during three years before first non-affective psychosis diagnosis | |
| Affective psychosis | F3 disorders with psychotic features: F30.2, F31.2, F32.3 F33.3 |
| Anxiety disorders | F40-41 |
| Bipolar disorder | F30-31 except F30.2 and F31.2 |
| Depression | F32-33 except F32.3 and F33.3 |
| Disorders of adult personality and behaviour | F60-69 |
| Substance use disorder | F10-F19 |
| Previous suicide attempt | X60-X84 and Y10-Y34 |
| **Other psychotropic medication use, measured during 3 months before first diagnoses (yes/no)** (between T-3 and T0 where 0 represents first admission/ visit with psychosis diagnosis) | |
| Antidepressants | N06A |
| Benzodiazepines | N05BA, N05CD |
| Z-drugs | N05CF |
| Other anxiolytics | N05B excluding N05BA |
| Other hypnotics | N05C excluding N05CD and N05CF |
| Mood stabilizers | N03AF01 carbamazepine, N03AG01 valproic acid, N03AX09 lamotrigine, N05AN01 lithium (any of these, yes/no) |

**Supplementary Table 2.** Description of the categorization of birth countries used throughout the study.

| Country Categories | *N (%)* | Description |
| --- | --- | --- |
| Afghanistan | 89 (0.69) |  |
| Iraq | 331 (2.55) |  |
| Iran | 169 (1.30) |  |
| Somalia  Sweden | 302 (2.33)  9927 (76.60) |  |
| Former Yugoslavia | 344 (2.65) | Bosnia-Hercegovina, Yugoslavia, Croatia, Macedonia, Serbia, Serbia and Montenegro, Slovenia |
| Other Africa | 356 (2.75) | Algeria, Angola, Arab republic Egypt, Burundi, Cameroon, Central African Republic, Djibouti, Egypt, Eritrea, Ethiopia, Gambia, Ghana, Guinea, Ivory Coast, Kenya, Kongo, Kongo Democratic Republic, Liberia, Libya, Malawi, Mali, Mocambique, Morocco, Nigeria, Rwanda, Senegal, Sierra Leone, Sudan, Somalia, South Africa, Swaziland, Tanzania, Togo, Tunisia, Uganda, Zimbabwe |
| Other Europe | 589 (4.54) | Albania, Armenia, Austria, Azerbaijan, Belarus, Belgium, Bulgaria, Cyprus, Czechoslovakia, Czech Republic, Denmark, Estonia, Finland, France, Georgia, Germany, Great Britain and Northern Ireland, Greece, Hungary, Iceland, Ireland, Italy, Kosovo, Latvia, Lithuania, Moldova, Netherlands, Norway, Poland, Portugal, Romania, Russia, Slovakia, Soviet Union, Spain, Switzerland, Ukraine |
| Other Middle East | 243 (1.88) | United Arab Emirate, Bahrain, Gaza area, Israel, Jordan, Kuwait, Lebanon, Palestine, Saudi Arabia, Syria, Turkey, Yemen |
| Asia | 367 (2.83) | Bangladesh, China, Philippines, India, Indonesia, Japan, Kazakhstan, Kirgizstan, Laos, Mongolia, Myanmar, Nepal, Pakistan, South Korea, Singapore, Sri Lanka, Taiwan, Thailand, Turkmenistan, Uzbekistan, Vietnam, Vietnam rep, Australia, Kiribati |
| The Americas | 243 (1.88) | Argentina, Bolivia, Brazil, Canada, Cuba, Chile, Colombia, Ecuador, El Salvador, Guatemala, Guyana, Haiti, Mexico, Nicaragua, Peru, The Dominican Republic, Uruguay, USA, Trinidad & Tobago, Venezuela |
